# Environmental Attitudes and (In)Actions Through Time: Older Adults’ Perspectives in Italy and Sweden

**DOI:** 10.1093/geroni/igaf122.1268

**Published:** 2025-12-31

**Authors:** Federica Previtali, Malin Bäckman, Virpi Timonen

**Affiliations:** University of Helsinki, Helsinki, Uusimaa, Finland; University of Helsinki, Helsinki, Uusimaa, Finland; University of Helsinki, Helsinki, Uusimaa, Finland

## Abstract

The climate crisis compels reflection on the future and the environments left for future generations. While public discussions often focus on youth as drivers of change, ageing societies bring forth the crucial role of the growing older population in addressing environmental issues. Later life can serve as a space for exploring tensions between environmental concerns, generational responsibilities, and related (in)actions. Later life is a period of time-related tensions: while a shrinking future time perspective may lead to a focus on individual goals, the ecological generativity theory highlights that transcendent goals gain prominence, extending beyond family legacies to include involvement in ecological activities. To explore these interrelated topics, we analyse 30 in-depth, semi-structured interviews with older adults (aged 60 and over) conducted in Italy and Sweden as part of the Legacies project, using a Constructivist Grounded Theory (CGT) approach. The analysis reveals the complexity surrounding environmental engagement throughout individual life courses, shaped by a caring relationship with the environment, access to ecological practices, concerns for grandchildren and intergenerational contact. By looking at older adults’ lived experiences and future-oriented perspectives, this study reflects dynamic positioning along a continuum between activism and cynicism in response to environmental crises in daily life.

